# Identification and Validation of Three Autophagy-Related Long Noncoding RNAs as Prognostic Signature in Cholangiocarcinoma

**DOI:** 10.3389/fonc.2021.780601

**Published:** 2021-12-02

**Authors:** Ya Jun Liu, Alphonse Houssou Hounye, Zheng Wang, Xiaowei Liu, Jun Yi, Min Qi

**Affiliations:** ^1^ Department of Gastroenterology, Xiangya Hospital Central South University, Changsha, China; ^2^ School of Mathematics and Statistics, Central South University, Changsha, China; ^3^ Information Science and Engineering School, Hunan First Normal University, Changsha, China; ^4^ Department of Plastic Surgery, Xiangya Hospital Central South University, Changsha, China

**Keywords:** autophagy, long noncoding RNAs, cholangiocarcinoma, The Cancer Genome Atlas, prognostic signature, Gene Expression Omnibus

## Abstract

Cholangiocarcinoma (CCA) is featured by common occurrence and poor prognosis. Autophagy is a biological process that has been extensively involved in the progression of tumors. Long noncoding RNAs (lncRNAs) have been discovered to be critical in diagnosing and predicting various tumors. It may be valuable to elaborate autophagy-related lncRNAs (ARlncRNAs) in CCA, and indeed, there are still few studies concerning the role of ARlncRNAs in CCA. Here, a prognostic ARlncRNA signature was constructed to predict the survival outcome of CCA patients. Through identification, three differentially expressed ARlncRNAs (DEARlncRNAs), including CHRM3.AS2, MIR205HG, and LINC00661, were screened and were considered predictive signatures. Furthermore, the overall survival (OS) of patients with high-risk scores was significantly lower than that of patients with low scores. Interestingly, the risk score was an independent factor for the OS of patients with CCA. Moreover, receiver operating characteristic (ROC) curve analysis showed that the screened and constructed prognosis signature for 1 year (AUC = 0.884), 3 years (AUC =0.759), and 5 years (AUC = 0.788) presented a high score of accuracy in predicting OS of CCA patients. Gene set enrichment analysis (GSEA) revealed that the three DEARlncRNAs were significantly enriched in CCA-related signaling pathways, including “pathways of basal cell carcinoma”, “glycerolipid metabolism”, etc. Quantitative real-time PCR (qRT-PCR) showed that expressions of CHRM3.AS2, MIR205HG, and LINC00661 were higher in CCA tissues than those in normal tissues, similar to the trends detected in the CCA dataset. Furthermore, Pearson’s analysis reported an intimate correlation of the risk score with immune cell infiltration, indicating a predictive value of the signature for the efficacy of immunotherapy. In addition, the screened lncRNAs were found to have the ability to modulate the expression of mRNAs by interacting with miRNAs based on the established lncRNA-miRNA-mRNA network. In conclusion, our study develops a novel nomogram with good reliability and accuracy to predict the OS of CCA patients, providing a significant guiding value for developing tailored therapy for CCA patients.

## Introduction

Cholangiocarcinoma (CCA) is such a dangerous malignancy originating from biliary epithelium that carries increasing morbidity and mortality currently (1, 2). There is a great difficulty in the early diagnosis of CCA owing to the occult location of bile duct system anatomically, and hence a majority of patients may loss the opportunity of radical surgery. The major therapeutic approaches for its treatment include interventional therapy, radiotherapy, targeted therapy, etc., which, however, have a limited curative effect and can lead to poor prognosis of patients with CCA (3–5). For instance, Rizzo et al. demonstrated that adding EGFR-mAbs to gemcitabine-based first-line chemotherapy could not significantly improve the overall survival rate of patients with advanced CCA, nor the objective response rate, and even lead to hematological and cutaneous adverse drug events (6). In addition, a more recent study by Rizzo et al. revealed that the role of adjuvant systemic chemotherapy is still the object of debate and controversy in the medical community of resected biliary tract cancer (BTC) (7). Considering the absence of efficient diagnostic tools in the early stage and available therapeutics at present, patients who enter the advanced stage may have a low 5-year survival rate of <5% (8). Currently, some molecular markers have been confirmed to provide explanation for the poor prognosis and tumor progression of CCA. For instance, high EGFR expression may predict postoperative CCA recurrence independently (9), and inducible nitric oxide synthase (iNOS) is involved in the pathogenesis of CCA in an inflammation-dependent manner (5). Unfortunately, CCA is indeed a disease with strong genetic heterogeneity, and there is so far poor understanding of its molecular mechanisms, resulting in a relatively low application of the majority of the identified markers in clinical data. It in turn highlights the importance of clarifying potential molecular mechanisms and cellular signaling pathways of CCA as well as finding new biomarkers with prognostic value, so as to benefit early detection of CCA and improvement of its prognosis.

Despite an initial recognition as “transcriptional noise” due to the absence of protein-coding capacity, long noncoding RNAs (lncRNAs; >200 nucleotides in length) have now been widely accepted to be a series of RNA molecules with critical functions (10, 11). Large numbers of novel lncRNAs have been identified with the development of sequencing technologies. Based on their regulatory roles of gene expressions at transcriptional, posttranscriptional, and translational levels, lncRNAs play biological functions in many cellular activities (12, 13). It is now accepted commonly that tumor progression can be partially explained by abnormal expression or dysfunction of lncRNAs, highlighting their key roles in tumor diagnosis and prognosis prediction in the oncology field. Cheng et al. (14), for example, found that lncRNA AC125603.2 had a promoting role in the biological activities of colon cancer cells and predicted a poor prognosis of colorectal cancer. Jia et al. (15) confirmed that lncRNA AC005229.4 could be regarded as a prognostic biomarker of hepatoma. However, the mechanism of lncRNAs in tumors has not been fully clarified due to the complexity of tumor physiological mechanisms and individual differences. It remains to be improved with respect to the accuracy of lncRNAs in predicting cancer prognosis, and further systematic studies are required to identify and explain multiple lncRNAs.

Autophagy is the main metabolic pathway in cells. It can decompose damaged proteins and organelles for energy recycling, and can participate in aging and various physiological and pathological processes related to aging. Autophagy can participate in maintaining the stability of the internal environment of life, whose function depends largely on the involvement of autophagy-related signaling pathways (16, 17). Under normal conditions, autophagy provides necessary circulating metabolites for cell survival and maintains cell homeostasis. However, autophagy can be abnormally activated in human malignancies, and exert different roles in different stages of tumors (18, 19). Nowadays, the importance of autophagy-related pathways has been paid much attention to, with the aim to search for novel targets to formulate targeted therapies for tumors. For example, Hector collected clinical evidence of autophagy imbalance during CCA and found autophagy dysfunction in the initial stage of CCA development, accompanied by increased expressions of autophagy markers in established tumors and invasive phenotypes. Furthermore, autophagy regulators could promote CCA cell death and reduce its invasive ability (20). In addition, lncRNAs have been disclosed to be possibly responsible for the autophagy of tumor cells. For instance, Luan et al. (21) reported 10 autophagy-related lncRNAs (ARlncRNAs) in predicting the prognosis of glioma and in regulating glioma biology. Deng et al. (22) also reported the value of LINC01559 for reliable prognostic prediction and individualized therapy development of pancreatic cancer patients. Given the current clinical status of CCA and considering the critical roles of ARlncRNAs, it may be a valuable direction of research to explore the role of ARlncRNAs in CCA, and indeed, there is still few study related to this topic. Here, our study attempts to establish an ARlncRNA signature, with emphasis placed on the identification of potential ARlncRNAs and exploration of their clinical significance in CCA, so as to assist the prediction of CCA patients’ prognosis and facilitate future drug selection.

## Materials and Methods

### Data Acquisition

Data of sequencing and survival that were specific to CCA were acquired from The Cancer Genome Atlas (TCGA) dataset (https://portal.gdc.cancer.gov/, RNAseq, I llumina). These data consisted of 36 CCA tissues and 9 adjacent normal biliary tissues, which were used as the test set. Clinical data were also extracted from this database, including age, gender and pathological stages. Simultaneously, the Human Autophagy Database (HADb) was also searched through visiting https://www.Autophagy.lu/index.html, with 232 autophagy gene datasets obtained. GSE107943 was downloaded from the Gene Expression Omnibus (GEO) database and contained data on 57 patients with CCA and their associated clinical information, which was selected as the validation cohort in this experiment.

### Identification and Construction of ARlncRNAs in CCA and Normal Tissues

The transcriptome sequencing data consisted of the following two parts: (1) protein-encoding mRNA (including autophagy-related gene (ARG) expression data); and (2) lncRNA expression data. By using R language, EdgeR package was utilized to analyze the differentially expressed autophagy-related genes (DEARGs) and differentially expressed lncRNAs (DElncRNAs) (|log2 Fold Change (FC)| > 1, adjusted *p*-value <0.05). After screening in the former step, the “ggplot2” package was applied for generating volcano plots, with corresponding heatmaps plotted by using R heatmap package.

### Coexpression Network Construction

Our study constructed the gene coexpression network (Cytoscape 3.8.2) to further investigate the differentially expressed ARlncRNAs (DEARlncRNAs). Furthermore, the correlations of DEARGs with DElncRNAs in CCA and normal tissues were disclosed by using Pearson’s correlation analysis. DEARlncRNAs were confirmed from the screened DElncRNAs with Pearson’s correlation coefficient (PCC) |R2| >0.3 and *p* < 0.001. Moreover, the coefficient of variation (CV) was also selected to get more available information in gene coexpression network analysis. The formula of CV can be described as follows: CV = *σ*/*µ* (*σ* and *µ* standard deviation, and mean of the subject of interest, respectively).

### Construction and Validation of Prognostic DEARlncRNA Signatures

Firstly, the ARlncRNA expression matrix was integrated with survival data. Then, the “survival” R package was used to identify ARlncRNAs showing intimate association with the overall survival (OS), with *p* < 0.01 indicating a statistically significant difference. Subsequently, the significant OS-related ARlncRNAs were further screened based on LASSO regression analysis by the “glmnet” package to avoid excessive overfitting of the signature model. The optimal value of the penalization coefficient lambda (*λ*) was obtained through cross-validation with 1,000 iterations to prevent overfitting. Eligible lncRNAs with the greatest suitability for building the signature were screened out finally based on the generated minimum *λ*. Next, the ARlncRNAs obtained from LASSO regression analysis were involved in subsequent multivariate Cox regression analysis. The signatures were constructed through different combination of lncRNAs, accompanied by the calculation of the Akaike information criterion (AIC) value for each independent lncRNA. Afterwards, the optimal prognosis signature was generated according to the minimum AIC value which had the goodness of fit. Risk scores were calculated based on $RiskScore=\Sigma_{i=1}^{n}\;\beta_{i}gene_{i}(expression)$ , where, *β_i_* is the coefficient of each gene expression, and gene (expression) represents DEARlncRNA expression. Two subgroups were divided based on the median value of the calculated risk scores, those who had high scores were classified into the high-risk group, and those with low scores into the low-risk group. The survival analysis for the different groups was realized by using the Kaplan-Meier (K-M) survival curve analysis and log-rank test by using the “survminer” R package. In addition, the specificity and sensitivity of the constructed prognostic signature were further calculated based on the area under the dynamic time-dependent receiver operating characteristic (ROC) curve (AUC) and the concordance index (C-index).

### Analysis of Risk Scores and Clinical Characteristics of CCA Patients

Patients’ clinical characteristics from TCGA were integrated with the risk score file by using “ggplot2” package to determine the presence of significant differences in risk scores. Univariate and multivariate Cox regression analyses based on clinical characteristics were then made to clarify whether the DEARlncRNA could predict patient prognosis independently. Subsequently, K-M analysis was used to identify the existence of significant differences in OS between groups when both groups shared common clinical characteristics. Gene set enrichment analysis (GSEA) could identify the significant enrichment of target gene set in some functional pathways. In this study, functional annotation was realized on the basis of GO and KEGG enrichment analyses by using the “clusterProfiler” package with NES >1 and FDR <0.05 (*p* < 0.01) to benefit subsequent pathway analysis of target mRNAs. The principal component analysis (PCA) was utilized for evaluating samples and expression patterns between high- and low-risk groups. In order to further investigate the discrimination among the prognostic values, the signature was then involved in assessing the relationship of the expression patterns with OS in tumor and normal tissues.

### Nomogram Based on the Signature of DEARlncRNAs for Prognostic Prediction in CCA Patients

For a quantitative prediction of the survival probability (1, 3, and 5 years) of CCA patients, a prognosis nomogram was established using the ARlncRNAs identified in our study and the clinical factors based on risk scores and other clinical characteristics by using the “survival” and “rms” packages of R language. The consistency of the prediction and actual outcomes was assessed based on C-index and displayed by calibration curves. Finally, the AUC values were evaluated to be associated with the depiction of the ROC curves of the nomogram. All the analyses were conducted in the test and validation cohorts.

### Regulatory Network Construction

DIANA Tools Online Suite was used to further identify the miRNAs related to lncRNAs, with a threshold preset at 0.9. Moreover, for a better understanding of the interaction between lncRNAs and miRNAs, this regulatory network was constructed with Cytoscape (version 3.8.2).

### Predictive Efficacy of Immunotherapy With the Established Signature

By using “CIBERSORT,” our study measured the infiltration expressions of different immune cells (*n* = 22) in CCA. In addition, the correlation between risk score and targeted therapeutic molecules was assessed for further clarification of the clinical value of the signature we constructed.

### Clinical CCA Sample Collection

The clinical samples used for experimental validation were CCA surgery-treated patients in Xiangya Hospital of Central South University from July 2020 to July 2021. The CCA samples and paired adjacent samples were collected intraoperatively and frozen immediately in liquid nitrogen for subsequent storage at −80°C. None of the patients received preoperative anticancer treatment. Written consent from each patient was provided before the surgery, with approval obtained as well by the Ethics Committee of our hospital.

### Quantitative Real-Time PCR

Collected tissues were subjected to the extraction of total RNA using Total RNA Kit II (Omega BioTek, Norcross, GA, USA). The quantity and quality of RNA were assessed by ultraviolet absorption spectrometry. Based on the manufacturer’s instructions, cDNA was synthesized from total RNA (1 µg) using the Quantscript RT Kit. qPCR was performed using the iQ SYBR Green Supermix on a CFX96 System (Bio-Rad Laboratories Inc., Hercules, CA,USA). Products were amplified using primers that recognized MIR205HG [ATCTCTCAAGTACCCATCTTGGA (forward); GGCCTCATGGTTGTCAGCTC (reverse)], LINC00661 [CTGTCCTGCGTACCTCCTCTGG (forward), CACTGCCTGCTGAGAAGTTGGATG (reverse)], CHRM3.AS2 [CATGCTGGCTGTGCTAGTTCTATCC (forward); GGCCCGTGATAATTCTCAGCAGAAC (reverse)], and GAPDH [CGTGCCGCCTGGAGAAACCTG (forward), AGAGTGGGAGTTGCTGTTGAA (reverse)]. A threshold cycle value (Ct) of each gene was produced and normalized to corresponding GAPDH in the same sample by processing the raw fluorescence data. Identical results were obtained with at least three repeated procedures independently.

### Statistical Analysis

**Figure 1:**
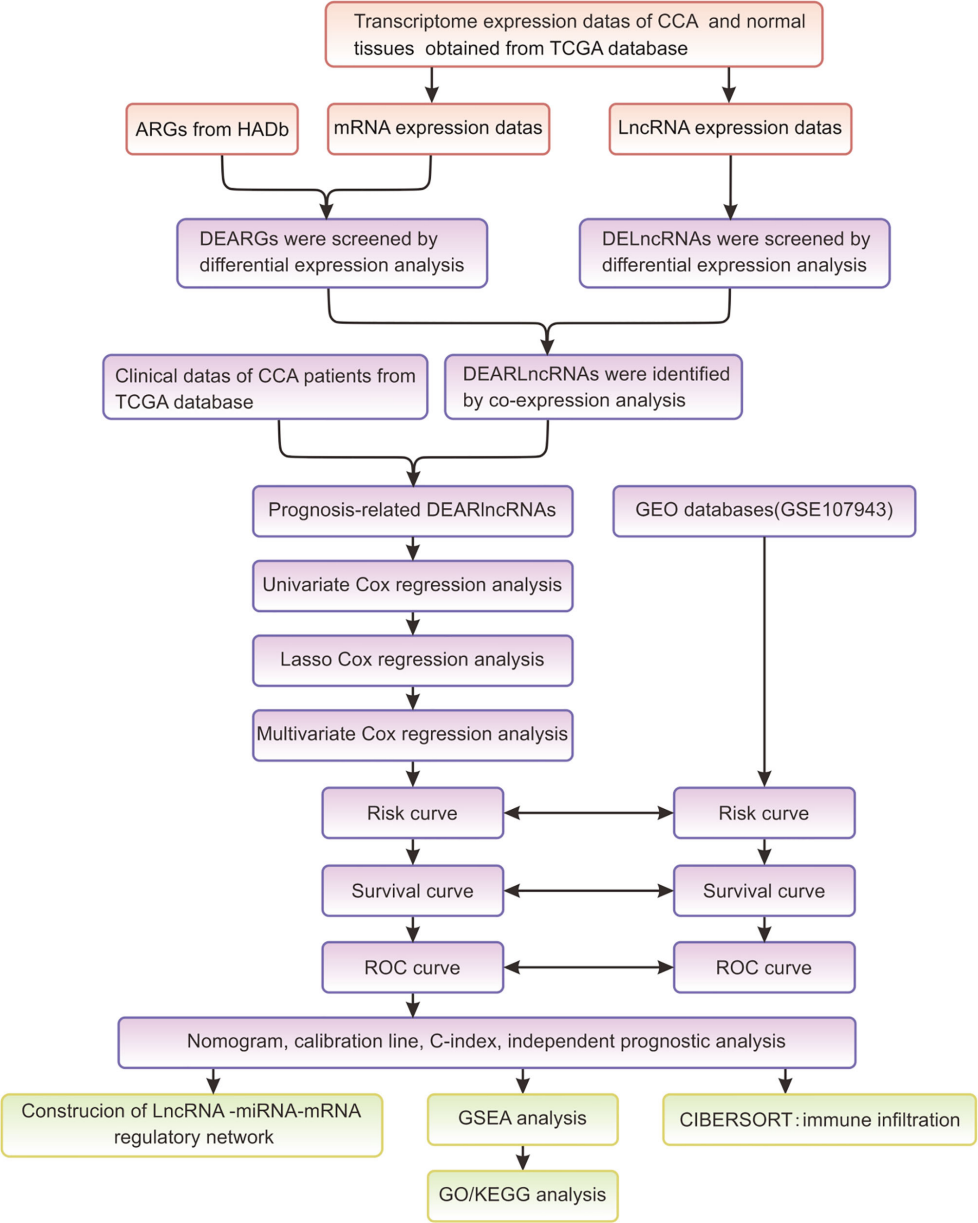
The flowchart of this study.

**Figure 1** summarized the flowchart of this study. R software (version 4.1.0) was the primary statistical tool for analysis. Cox regression analyses were performed for screening survival-related DEARlncRNAs and establishing the risk score model. K-M analysis was used for analyzing survivals, and the differences in survivals were evaluated by log-rank test. ROC curve and AUC were displayed by using “Survival ROC” package in R language. *p* < 0.05 was preset to determine the statistical difference during statistical analysis.

## Results

### Identification of DEARlncRNA Based on TCGA Data

**Figure 2:**
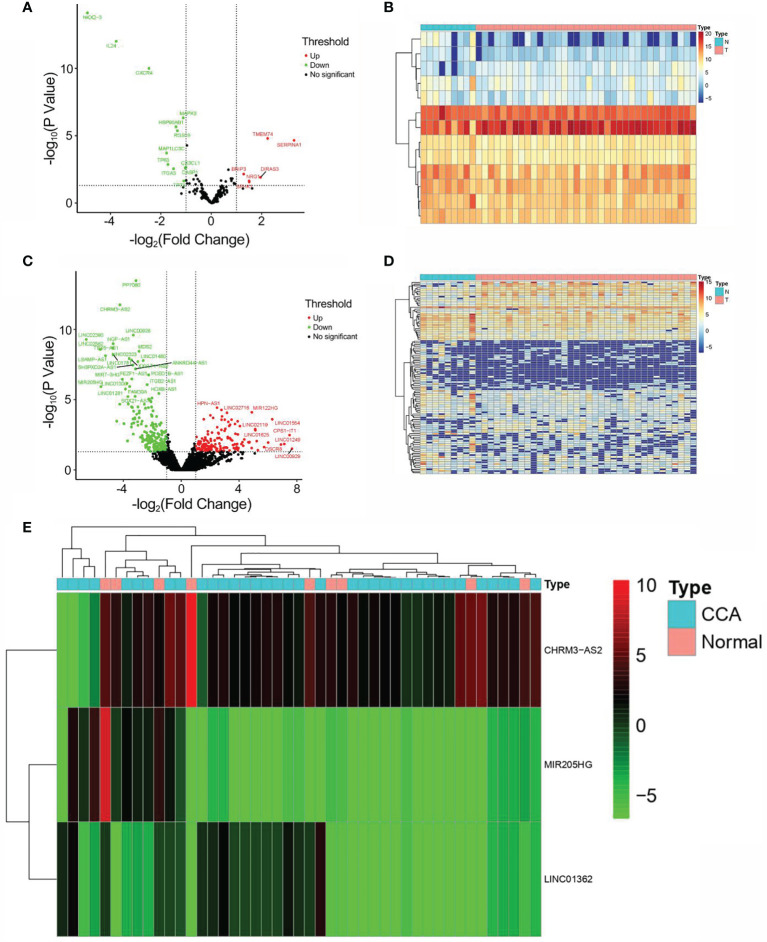
Identification of DEARGs and DElncRNAs. **(A)** Volcano plot of DEARG distribution (*n* = 13; red dots, upregulated; green dot, downregulated). **(B)** Heatmap of DEARG expression profiles. **(C)** Volcano plot of DElncRNA distribution (*n* = 108; red dots, upregulated ARGs; green dot, downregulated ARGs). **(D)** Heatmap of DElncRNA expression profiles. **(E)** Heatmap of the expression profiles of CHRM3.AS2, MIR205HG, and LINC00661 in CCA patients and normal controls.

Through searching TCGA, RNA-seq and clinical follow-up data (tumor, *n* = 36; normal, *n* = 9) were obtained as the test set. Among the 232 ARGs downloaded from HADb, there were 219 DEARGs in CCA. Consequently, a total of 13 DEARGs and 108 DElncRNAs were screened (|log2F C| >1 and FDR <0.05). The expressions of DEARGs and DElncRNAs between CCA and normal tissues were then identified based on the plotted volcano plot and heatmap (**Figures 2A–E**). A total of 92 DElncRNAs were identified to be statistically significant (PPC >0.3 and *p* < 0.001) and were hence selected as the DEARlncRNAs for subsequent validation.

### Establishment of Prognostic DEARlncRNA Signature for CCA Patients

**Figure 3:**
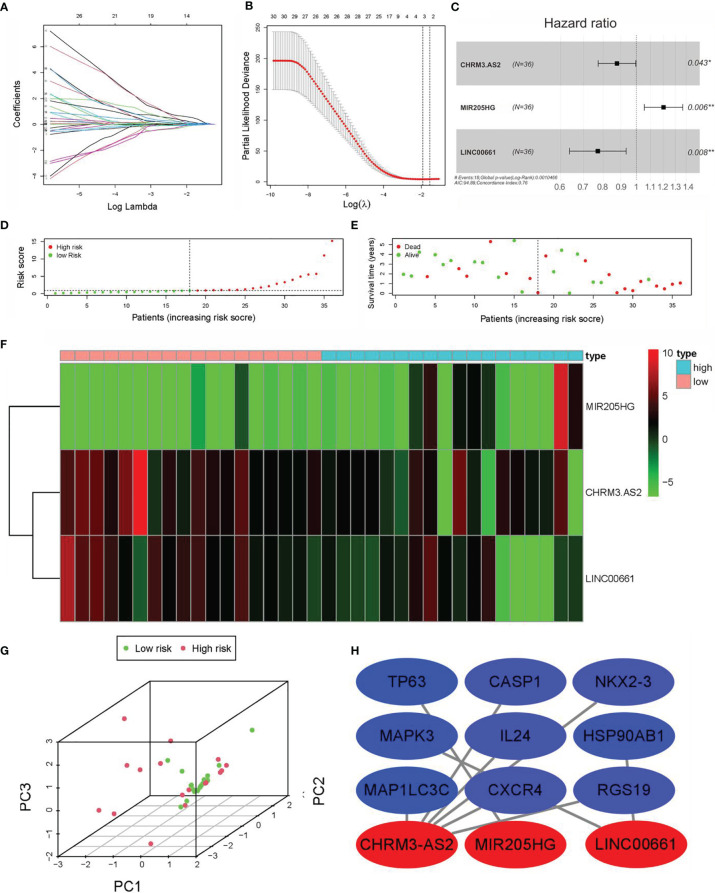
Construction of prognostic DEARlncRNA signatures for CCA patients based on TCGA data. **(A)** univariate Cox regression analysis. **(B)** LASSO-penalized Cox regression analysis. **(C)** Multivariate Cox regression analysis. **(D)** Distribution of CCA patients with high and low risk based on TCGA data. **(E)** Survival status of CCA patients with high and low risk based on TCGA data. **(F)** The heatmap of the three DEARlncRNAs expression profiles in high- and low-risk CCA patients. **(G)** PCA of the expression profiles of CHRM3.AS2, MIR205HG, and LINC00661. **(H)** Coexpression relationship of CHRM3.AS2, MIR205HG, and LINC00661 with corresponding ARGs (*P < 0.05, **P < 0.01).

As shown in **Figure 3A**, 59 lncRNAs were further identified from the 92 DEARlncRNAs screened above (all *p* < 0.05). When minimum *λ* = 0.0345, four DEARlncRNAs were obtained, which could reduce the overfitting of the signature (**Figure 3B**). Based on AIC = 94.89, these DEARlncRNAs were then selected for multivariate analysis. Finally, three DEARlncRNAs (CHRM3.AS2, MIR205HG, and LINC00661) were identified (**Figure 3C**) for subsequent construction of a predictive model. Furthermore, MIR205HG was identified to have a high hazard ratio (HR = 1.055, *p* = 0.0056) and was defined as high-risk factor, whereas CHRM3.AS2 and LINC00661 were identified to have low hazard ratios (HR = 0.877, *p* = 0.0432 and HR = 0.771, *p* = 0.008) and were defined as low-risk factor. A formula of the risk model was established by exploring the best three DEARlncRNAs based on the prognosis signature. The formula can be given as follows: Risk score = −(0.1309 * CHRM3.AS2) + (0.1833 * MIR205HG) − (0.2603 * LINC00661). The risk score of each patient was then determined on the basis of the detected expressions of CHRM3.AS2, MIR205HG, and LINC00661. As described previously, patients (test set) were grouped into high-risk (*n* = 18) and low-risk (*n* = 18) groups when median risk score = 0.896. **Figure 3D** shows a gradual elevation of the score from left to right. **Figure 3E** displays the survival of each CCA patient. **Figure 3F** shows the heatmap of CHRM3.AS2, MIR205HG, and LINC00661 expression profiles in both groups (**Figure 3F**). Furthermore, compared with low-risk group, the three DEARlncRNAs were observed to be highly expressed in the high-risk group, and corresponding expression profiles had differences significantly, as evidenced by PCA in **Figure 3G**. Moreover, coexpression network analysis revealed the relationship between DElncRNAs and DEARGs with consistent prognosis signature using the threshold PCC >0.3 and *p* < 0.001 (**Figure 3H**).

### Verification of the Predictive Ability of the Three DEARlncRNA Prognostic Signatures

**Figure 4:**
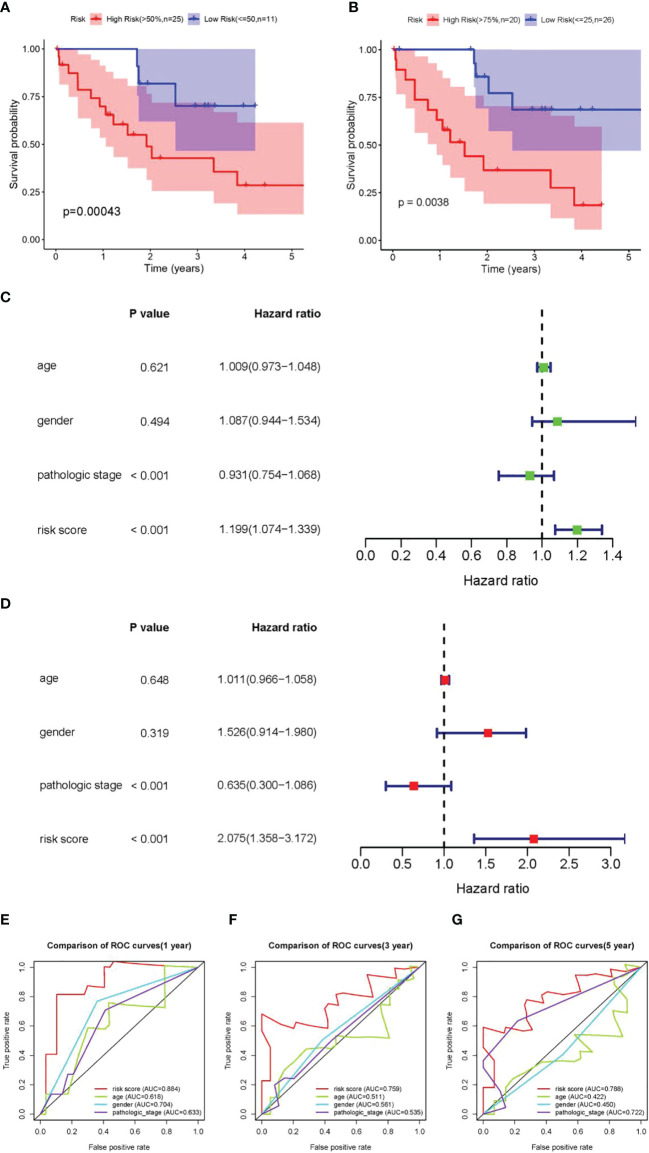
Validation of the prognostic DEARlncRNA signatures of CHRM3.AS2, MIR205HG, and LINC00661. **(A)** The K-M curve analysis in high-risk (>50%) and low-risk (≤50%) patients. **(B)** The K-M curve analysis in high-risk (>75%) and low-risk (≤25%) patients. **(C)** Relationship of clinical characteristics and risk scores with OS of CCA patients presented by forest plot. **(D)** Relationship of clinical characteristics and risk scores with OS of CCA patients presented by forest plot. The 1-year (AUC = 0.884) **(E)**, 3-year (AUC = 0.759) **(F)**, and 5-year (AUC = 0.788) **(G)** time-dependent ROC curves.

Further verification was promoted to confirm the predictive ability of the three DEARlncRNAs identified above. Firstly, patients in the high-risk group were observed to have shorter OS time than those in the low-risk group, as revealed by K-M analysis in **Figure 4A**, similarly to the trends described before. With another validation of the role of risk scores, patients were then divided based on the quartiles (*Q*). Again and similarly, a worse OS was noticed in those with high scores relative to those with low scores (**Figure 4B**). Furthermore, as presented in **Figures 4C, D**, both pathological stage and risk scores were confirmed to be effective prognosis factors for CCA patients (*p* < 0.001). Moreover, the ROC curve analysis showed a high prediction accuracy of patient survival, demonstrating good agreement, sensitivity, and specificity of the risk score. The AUC of 1-, 3-, and 5-year time-dependent ROC curves were 0.884, 0.759, and 0.788, respectively (**Figures 4E–G**).

### Verification of the Predictive Ability of Prognostic Signatures in the Test Set (GSE107943)

**Figure 5:**
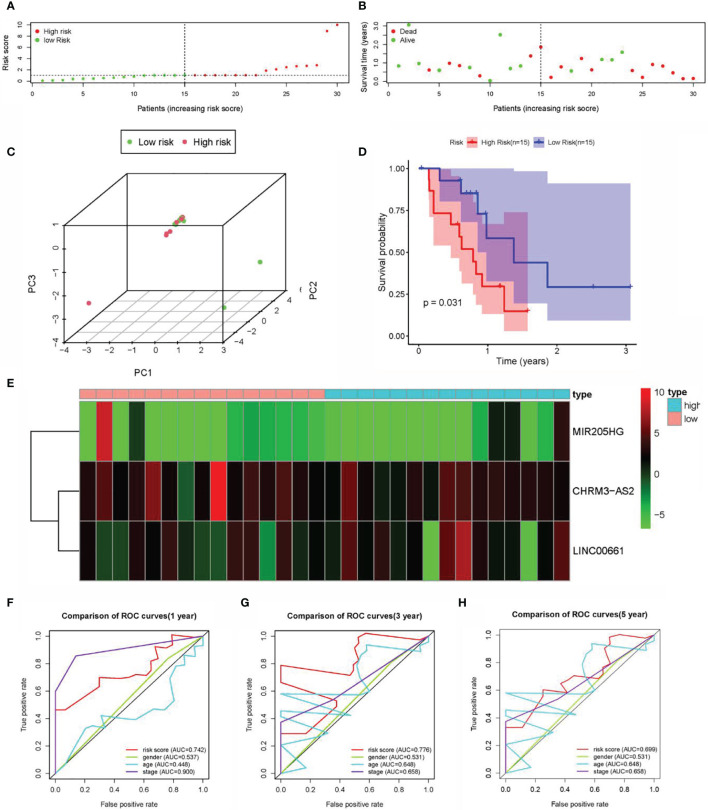
Construction of the prognostic DEARlncRNA signatures for CCA patients using GEO datasets. **(A)** Distribution of CCA patients with high- and low-risk scores. **(B)** Survival status of CCA patients with high- and low-risk scores. **(C)** PCA of the expression profiles of CHRM3.AS2, MIR205HG, and LINC00661. **(D)** The K-M curve analysis in high-risk (*n* = 15) and low-risk (*n* = 15) patients. **(E)** The heatmap of the expression profiles of CHRM3.AS2, MIR205HG, and LINC00661. **(F)** The 1-year time-dependent ROC curve. **(G)** The 3-year time-dependent ROC curve. **(H)** The 5-year time-dependent ROC curve.

For the validation of the predictive power of CHRM3.AS2, MIR205HG, and LINC00661, dataset GSE107943 containing 57 samples (tumors, *n* = 32 and normal tissues, *n* = 25) was used for validation. Based on the same processing on the TCGA database, 30 samples were obtained after combining the DEARlncRNAs with clinical follow-up data. Two groups were also set, with 15 cases in each group (median risk score = 1.031). **Figures 5A, B** shows the distribution of the risk score and survival status of each patient. The results were consistent with that obtained on the TCGA database. However, neither the heatmap nor the PCA showed a clear distinction between patients with high- and low-risk scores (**Figures 5C, E**), which was possibly attributed to the limited sample size of the available TCGA dataset. Fortunately, patients in the high-risk group (*n* = 15) were observed to have shorter OS time than those in the low-risk group (*n* = 15) by K-M analysis, supporting the predictive power of the proposed signature (**Figure 5D**). Further ROC curve analysis revealed that the AUC for 1-, 3-, and 5-year time-dependent ROC curve were 0.742, 0.776, and 0.699, respectively (**Figures 5F–H**), confirming the consistency described on CCA datasets in the TCGA database (**Figure 5C**).

### Correlation Analysis of the Risk Scores With Clinical Characteristics of CCA Patients

**Figure 6:**
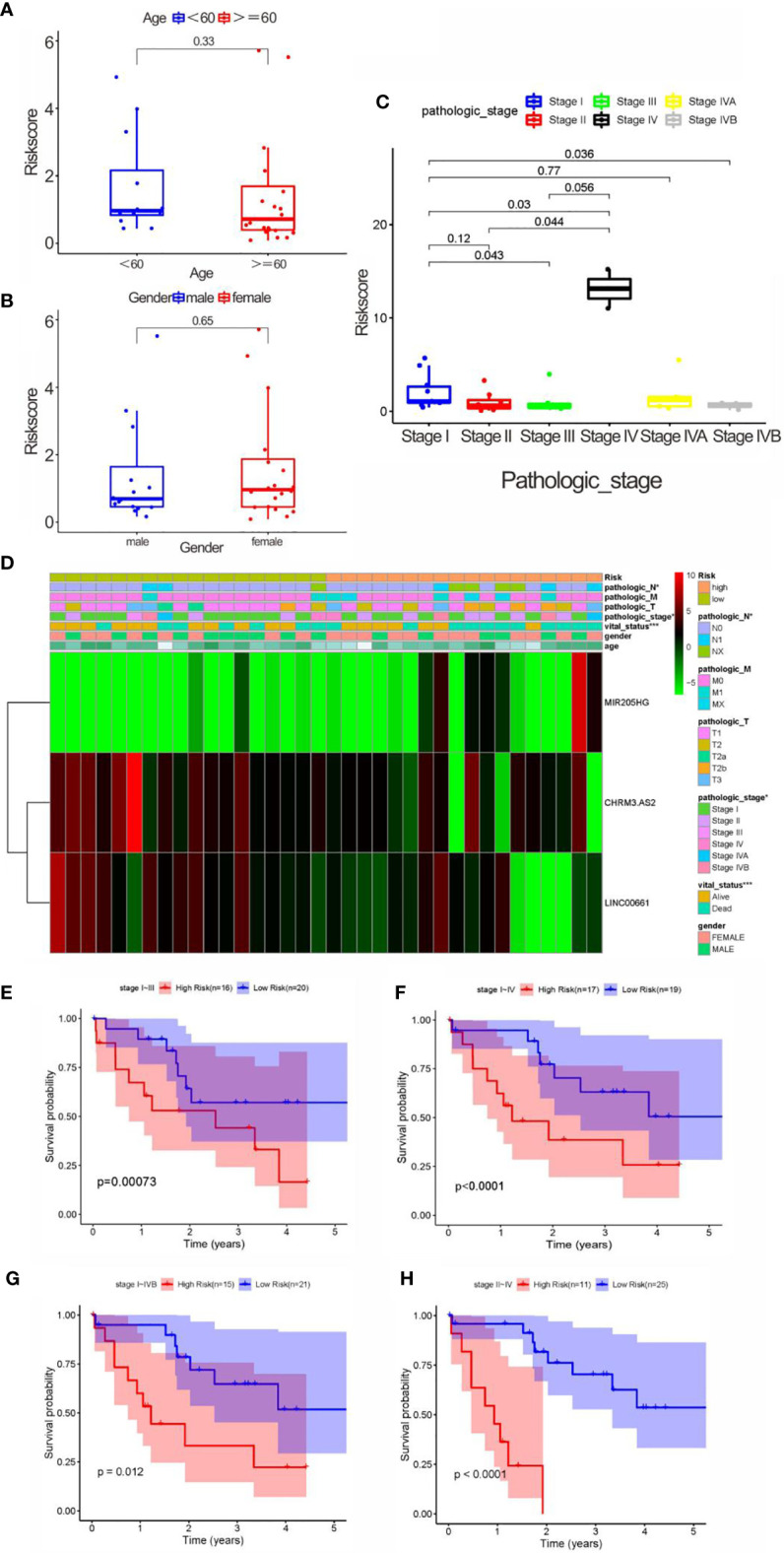
Correlation analysis between clinical characteristics and risk scores. **(A)** Correlation between age and risk score, without significant difference (<60, and 60 years old). **(B)** Correlation between gender and risk score, without significant difference. **(C)** The correlation between pathological stage and risk score. **(D)** Heatmap of three key prognostic DEARlncRNA in the correlation of risk group and clinical traits. **(E)** K-M curve based on pathological stage (stages I and III). **(F)** K-M curve based on pathological stage (stages I and IV). **(G)** K-M curve based on pathological stage (stages I and IVB). **(H)** K-M curve based on pathological stage (stages II and IV).

We firstly compared the impact of clinical characteristics on each patient with CCA in high- and low-risk groups. Despite with no obvious correlation found with gender and age (both *p* > 0.05) (**Figures 6A, B**), the risk score indicated an evident correlation with pathological grade (e.g., stage I vs. stages III–IV; stage II vs. stage IV) (both *p* < 0.05) (**Figures 6E–H**). These results showed that there might be a higher risk score as the pathological grade increased, which might indicate a worse prognosis. However, no statistical difference was noticed in patients with stage II vs. stage III (*p* > 0.05). Furthermore, the high-risk score was significant correlations with pathological stage, N stage, and survival status (**Figures 6C, D**).

### Construction and Validation of the Nomogram

**Figure 7:**
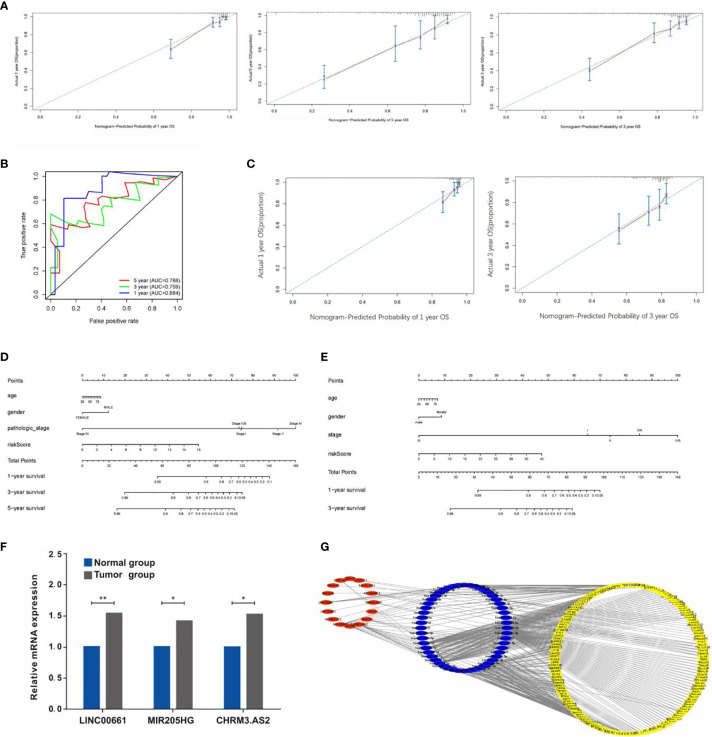
**(A)** Calibration plots of the predictive accuracy of the nomogram for 1, 3, and 5 years in TCGA data. **(B)** Time-dependent ROC curve in TCGA data. **(C)** Calibration plots based on the GEO data. **(D)** Nomogram based on the signature and clinical information in the TCGA data. **(E)** Nomogram based on the signature and clinical information in the GEO data. **(F)** qRT-PCR detection of the expressions of LINC00661, MIR205HG, and CHRM3.AS. **(G)** Construction of lncRNA-miRNA-mRNA regulatory network (*P < 0.05, **P < 0.01).

A nomogram was established and validated using data from TCGA (**Figure 7D**) and GEO (**Figure 7E**) respectively to determine the survival rate of CCA patients conveniently. As a result, the calibration plots had excellent prediction accuracy, showing an approximately similar trend of the predicted survival to that of the actual results (**Figure 7A**). Furthermore, the ROC curve confirmed that the predictive ability of the nomogram has good accuracy for 1-, 3-, and 5-year OS, with corresponding AUC of 0.884, 0.759, and 0.788, respectively (**Figure 7B**). Interestingly, the calibration curve of the nomogram based on GEO data also demonstrated a good accuracy of the 1- and 3-year predictive survival rates (**Figure 7C**). Calibration curves showed that the nomogram had a superior agreement between the predicted and actual OS in both cohorts (**Figures 7D, E**).

### Validation of the Expressions of LncRNAs in CCA Samples

As mentioned previously, the expressions of CHRM3.AS2, MIR205HG, and LINC00661 from the TCGA and GEO databases were remarkably upregulated in tumor tissues compared with those in normal tissues. In view of the above results, six paired CCA samples and matching adjacent nontumor tissues obtained clinically were used for examining the mRNA levels of CHRM3.AS2, MIR205HG, and LINC00661 *via* quantitative real-time PCR (qRT-PCR). Corresponding results were consistent with the trends reported based on data from the TCGA and GEO databases (**Figure 7F**) (all *p* < 0.05).

### Regulatory Network Construction

LncRNAs could have a regulatory role in the biological features of cancers based on a network of lncRNA-miRNA-mRNA through interacting with miRNAs to modulate mRNA expression, and hence mediating the initiation of malignant tumor development. In order to explore the regulation of these screened lncRNAs, our study further established a regulatory network including 16 lncRNAs, 52 miRNAs, and 156 mRNAs (**Figure 7G**).

### Functional Analysis of the Signature

**Figure 8:**
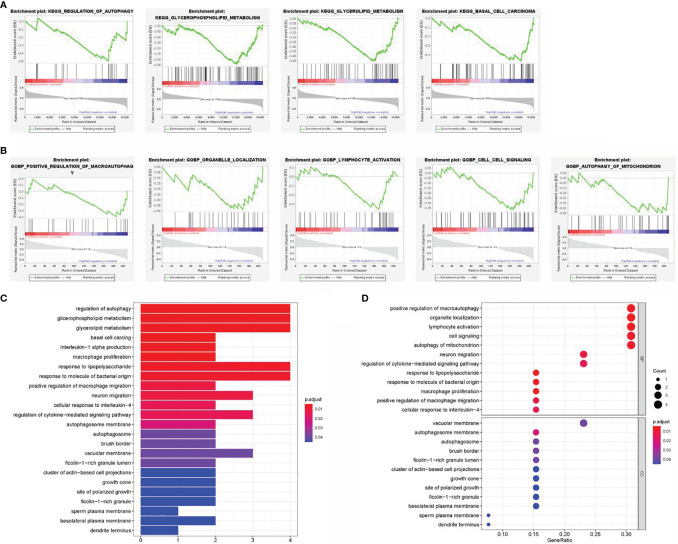
Gene set enrichment analysis (GSEA) of the high- and low-risk groups. **(A)** Enrichment analysis of KEGG pathway. **(B)** Enrichment analysis of GO pathway. **(C)** Barplot graph for KEGG pathways. **(D)** Bubble graph for GO enrichment.

A hypothesis was proposed that the predictive performance of the constructed prognostic DEARlncRNA signature based on CHRM3.AS2, MIR205HG, and LINC00661 relied on the biological functions of lncRNAs in CCA. In order to explore the potential mechanism, GSEA was performed to identify the enrichment of KEGG and GO pathways in the high-risk group. In **Figures 8A, C**, the top 4 KEGG pathways were “pathways of basal cell carcinoma”, “glycerolipid metabolism”, “glycerophospholipid metabolism”, and “regulation of autophagy”, respectively. Moreover, five GO signaling pathways were significantly altered (**Figures 8B, D**), including “positive regulation of macroautophagy”, “organelle localization”, “lymphocyte activation”, “cell signaling”, and “autophagy of mitochondrion”.

### The Relationship of Signature and Immunity in CCA Tissues

**Figure 9:**
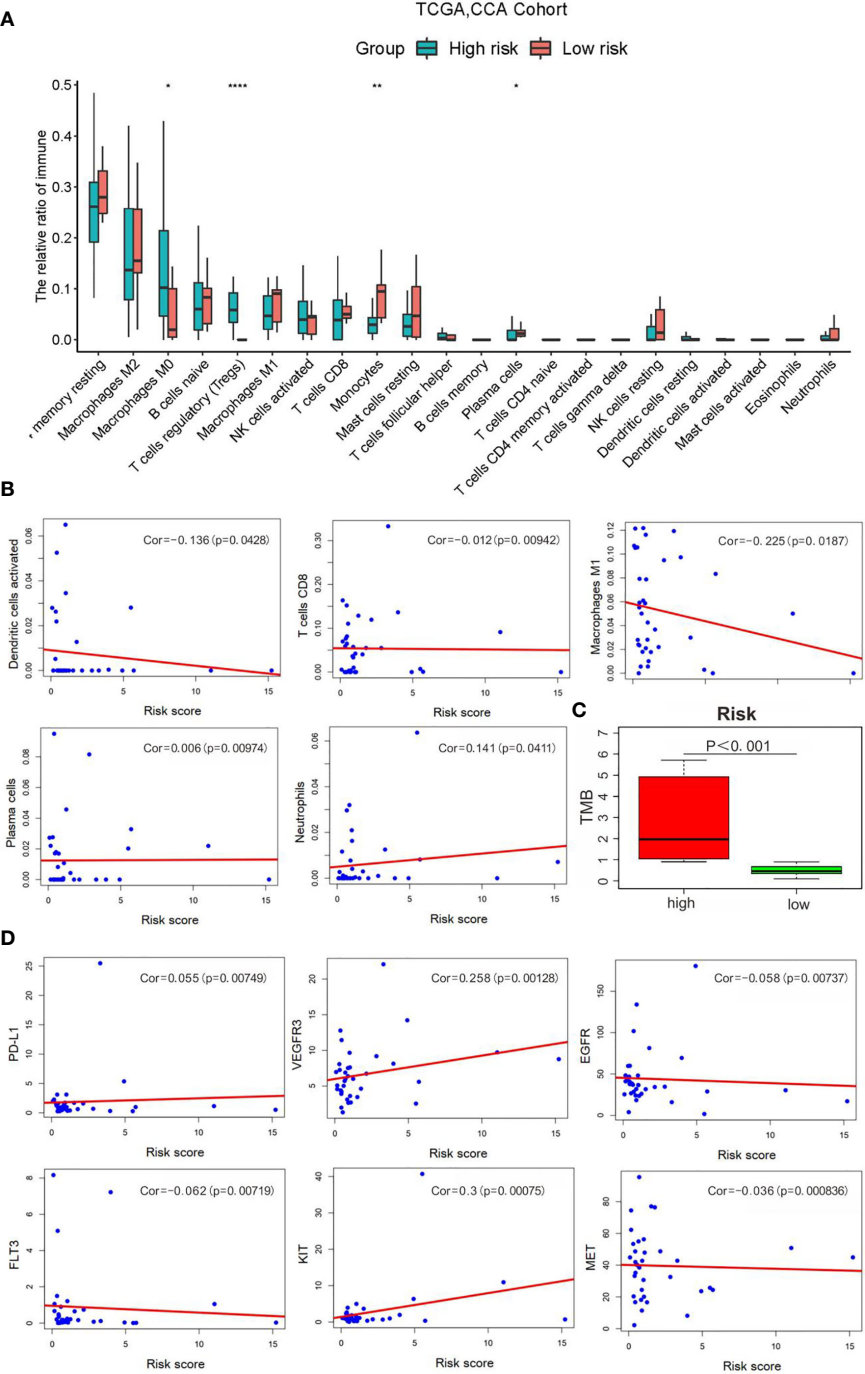
Correlation analysis of risk scores with the clinical characteristics, immune cells and therapy targets. **(A)** Relative infiltration expression of 22 immune cells between different risk groups. (*P < 0.05, **P < 0.01, ****P < 0.0001); **(B)** Correlation of with immune cells; **(C)** TMB evaluation; **(D)** Correlation with therapy targets.

It is common knowledge that the tumor mutation burden (TMB) can be associated with the clinical efficacy of immunotherapy (23). **Figures 9A, B** shows that macrophages M0, T-cell regulatory (Tregs), and plasma cells were increased evidently in the high-risk group, yet with an obvious decrease in monocytes and other protective immune cells. Accordingly, our study evaluated the TMB of CCA patients to explore the value of the signature established in our study for efficacy prediction. As shown in **Figure 9C**, patients in high-risk group had higher TMB, implying the potential effective outcome of immunotherapy. Furthermore, a close correlation of the score was found with PD-L1 (cor = 0.055, *p* = 0.0074), VEGFR3 (cor = 0.258, *p* = 0.00128), EGFR (cor = −0.058, *p* = 0.00737), FLT3 (cor = −0.062, *p* = 0.0072), KIT (cor = 0.3, *p* = 0.00075), and MET (cor = −0.036, *p* = 0.00083) (**Figure 9D**).

## Discussion

CAA has been recognized as the second most commonly diagnosed primary liver tumor (24). It results from cholangiocyte differentiation and can be developed from any part of the biliary tree (25). Current data support that it has rising morbidity and mortality, difficulty in the early diagnosis, and unsatisfied therapeutic outcome, resulting in a poor prognosis of this cancer that has attracted the special attention of the medical community (1, 26, 27). Its overall 5-year survival is estimated to be less than one-third in patients undergoing radical surgery (28). The etiology of CCA is related to a strong genetic heterogeneity, and there is an absence of comprehensive cognition of the pathogenesis of CCA at present (29, 30). It is still controversial with regard to the genetic changes involved in CCA initiation, progression, and prognosis. At present, there is a relatively low clinical applicability of the available biomarkers although they are valuable to predict, diagnose, or provide a therapeutic effect on CAA, e.g., mucin antigen MUC1, fascia, and EGFR (31–33). So far, there are still many gaps in the research of valuable biomarkers for CAA with high practicability. As we have described before, autophagy has a role in CCA, and there is a dysfunction of autophagy in the initial stage of CCA development. In addition, autophagy modulators can promote CCA cell death and reduce the invasiveness capacity of tumor cells (34, 35). Thus, there may be a great significance to identify potential autophagy-related molecules for predicting CCA prognosis. Here, on the basis of development in genomics, our study constructed a reliable prognostic ARlncRNA signature for CCA.

In this study, the TCGA and GEO were retrieved for data collection to explore the prognosis of ARlncRNAs for CCA. Our study initiated from the analysis of the transcriptome and clinical data of CCA patients in TCGA, followed by identifying 108 ARlncRNAs through the lncRNA-autophagy gene coexpression analysis. Subsequently, a signature based on CHRM3.AS2, MIR205HG, and LINC00661 was constructed for OS prediction. The constructed prognosis signature was useful for risk score calculation separately for each case, which was evaluated in the test set. Our study indicated a low survival and thus a worse prognosis in patients with high-risk scores. The detection results were found to be similar with those in validation cohort. Meanwhile, the risk score was also confirmed to have a significant correlation with tumor pathological grade. It is speculated that patients with higher tumor pathological grades may have higher scores and thus poor prognosis. Therefore, a hypothesis was proposed in our study that the prognostic DEARlncRNA signatures of CHRM3.AS2, MIR205HG, and LINC00661 might be responsible partially for CCA progression. Further analysis of the 1-, 3-, and 5-year time-dependent ROC curves of CCA patients revealed that the prognostic signature was found to have good sensitivity and specificity, suggesting good reliability in prediction. Furthermore, a nomogram was established, and the calibration plot showed that the predicted survival was in good agreement with that of the actual situation, which in turn confirms the good predictive performance of the nomogram constructed in our study. Finally, the expressions of CHRM3.AS2, MIR205HG, and LINC00661 were identified in clinical samples, with similar trends based on database analyses. Collectively, CHRM3.AS2, MIR205HG, and LINC00661 may be considered wonderful predictors in CCA prognosis and can be regarded as powerful indicators for patients with CCA in clinical practice.

The function of autophagy in reducing DNA damage and oxidative stress in cells in the early stage of tumors is well known. Nevertheless, autophagy can also promote tumor progression by providing sufficient energy to tumor cells under various adverse environments. Whereas, the pathological role of autophagy and its therapeutic potential in CCA are still unclear. Importantly, the prognostic significance of autophagy-related markers, emphasizing the importance of this process in tumors has been identified. It has been documented that regulating autophagy-related signaling pathways, such as PI3K/Akt/mTOR, p53and JAK/STAT, can significantly affect epithelial-mesenchymal transition, which may be drivers of tumor aggravation, and thus may result in adverse outcomes in tumor growth and metastasis, and even drug resistance (36). Moreover, past research has verified the potential prognostic value of Beclin1 for CCA (37). Interestingly, Beclin1 plays an important role in linking autophagy, apoptosis, and differentiation. In addition, He et al. demonstrated that cellular autophagy can be promoted through modulating FOXO1 expression and transcriptional activity. Through acetylation, FOXO1 can interact with ATG7 to regulate basal and starvation-induced autophagy in CCA cells (38). The present study intended to construct a prognostic DEARlncRNA signature for CCA in view of the importance of autophagy and the lack of study of related lncRNAs for the disease we studied.

Among the three selected DEARlncRNAs, Yan et al. identified the prognostic significance of CHRM3.AS2 in ovarian carcinoma, with possible association with hedgehog pathway, basal cell carcinoma, Wnt signaling pathway, etc. (39). Interestingly, CHRM3.AS2 was a risk-associated gene in our study, which was highly expressed in CCA. Moreover, as for MIR205HG, Li et al. demonstrated that MIR205HG could exert roles on cell cycle, migration, and apoptosis of esophageal squamous cell carcinoma by mediating miR-214 negatively and regulating SOX4 as a molecular sponge, which can be regarded as a novel candidate for the diagnosis and treatment of that tumor (40). In another recent study, Liu and colleagues demonstrated that by acting as a competing endogenous RNA, MIR205HG could accelerate lung squamous cell carcinoma development *via* mediating the molecular axis of miR-299-3p/MAP 3 K2 (41). Furthermore, MIR205HG could also target SRSF1 and modulate KRT17 to mediate biological activities of cervical cancer cells (42). In our study,the expressions of CHRM3.AS2, MIR205HG, and LINC00661 were significantly increased in tumor tissues of CCA patients, further verifying the prognostic prediction value of the established signatures for CCA.

The effective prognostic prediction of the three ARlncRNAs could be interrelated with the biological functions of the lncRNAs in CCA. However, there is a lack of report on the biological functions of CHRM3.AS2, MIR205HG, and LINC00661 in our signature previously. Therefore, to determine the underlying mechanism, GSEA revealed patients with high-risk scores showed enrichment of “pathways of basal cell carcinoma”, 5“glycerolipid metabolism”, “positive regulation of macroautophagy”, and “organelle localization”. The significant enrichment results suggest a higher risk of developing CCA under the aforementioned conditions. These results indicate the association of high scores with autophagy modulation, and also provide potential therapeutic targets for patients with CCA. Furthermore, autophagy is complicated with the involvement of multiple ARGs and signaling pathways, forming a huge and complex regulatory network to mediate the activities of tumor cells. Hence, a lncRNA-miRNA-mRNA regulatory network was constructed in our study that benefits the understanding of the potential biological mechanism of ARlncRNAs. In addition, immunotherapy may be effective for treating CCA patients with high-risk scores. Also, the risk score was positively correlated with macrophages M0, Tregs, and plasma cells, as suggested by the immune cell infiltration analysis. These findings suggested that these DEARlncRNA signatures screened in our study may be closely related to immune microenvironment and classical signaling pathways. Collectively, CHRM3.AS2, MIR205HG, and LINC00661 may regulate autophagy through the above various pathways, leading to differences in survival outcomes according to prognostic characteristics of CCA patients with high- and low-risk scores. Furthermore, similar studies may have been reported, for example, Cao et al. (43) reported most recently in May 2021 a similar exploration of DEG signature related to TME for CCA patients’ prognosis prediction. Their study emphasized on TME and DEGs, while our study focused on autophagy and DEARlncRNAs, both of which deserve affirmation with positive and promising results generated. Importantly, there are some highlights in this study; to be specific, our study discloses the significance of three ARlncRNAs with differential expressions in predicting the prognostic outcomes of CCA based on abundant data assessment, accompanied by experimental verification, which, of course, remains to be explored comprehensively in the future.

Inevitably, there are several limitations in our current study which shall be taken into consideration in a cautious manner. Firstly, the sample size of the CCA TCGA database is relatively small that may affect the reliability and accuracy of the predictive model, which constitutes the main disadvantage of this study. Secondly, some of the findings in our research were obtained based on speculation and assumptions from bioinformatics analysis, with lack of support from our own experiments and of larger sample scales for confirmation. Further confirmation based on our own sufficient experiments will contribute a lot to improve the reliability of our study, which is the major direction of our research. In addition, considering the emergence of bioinformatics analysis for the screening of molecular or drug targets and analysis of functional pathways, there may be some methodological overlaps. Anyway, findings based on these analyses are valuable for further screening and references on the basis of prospective analysis combined with retrospective designs jointly. Significantly and specifically, concerning the potential direction of research based on our exploration, the expression of the three DEARlncRNAs can be further knocked out in CCA cells *in vitro* by transfection technology to explore the effects of silencing the three DEARlncRNAs level on the proliferation, migration, and invasion of CCA cells. In addition, a syngeneic mouse model of CCA can be further established to analyze the expression of the three DEARlncRNAs in various carcinogenic development stages and their correlation with immunity, so as to further verify which autophagy markers can benefit from the inhibition or activation of autophagy. Finally, the roles of three DEARlncRNAs in autophagy, chemotherapy resistance, and targeted therapy are also worthy of further exploration, which is a promising strategy to improve the therapeutic expectation of CCA patients.

In conclusion, our study constructs an ARlncRNA coexpression network and identifies a signature of three DEARlncRNAs with prognostic value for CCA patients. This study also identifies and validates a novel and robust nomogram combining the signature and clinical characteristics to predict the 1-, 3-, and 5-year OS rates of CCA patients. Findings in this study may contribute to the formulation of individualized therapies for CCA patients and may provide a therapeutic reference for other tumors. Meanwhile, considering the existence of certain deficiencies in this analysis, further investigation is scheduled by our research team to confirm the exact roles of CHRM3.AS2, MIR205HG, and LINC00661 and corresponding utility in the clinical setting based on more *in vivo* and *in vitro* experiments rather than bioinformatic analysis primarily.

## Data Availability Statement

The datasets presented in this study can be found in online repositories. The names of the repository/repositories and accession number(s) can be found in the article/**Supplementary Material**.

## Ethics Statement

Ethics committee approval and written informed consents were obtained from participants. The study was performed based on standard principles of ethical principles and professional conduct.

## Author Contributions

YL drafted the manuscript. AH collected and carried out data. ZW and MQ designed the study and critically revised the manuscript. JY provided general advice and supervised the whole process of this study. All authors contributed to the article and approved the submitted version.

## Funding

This work was supported by the National Natural Science Foundation of China (No. 81770584 and No. 82000502), Natural Science Foundation of Hunan Province (No. 2020SK2068 and No. 2020JJ5941), and the Projects of the National Social Science Foundation of China (grant number No. 82073019 and No. 82073018).

## Conflict of Interest

The authors declare that the research was conducted in the absence of any commercial or financial relationships that could be construed as a potential conflict of interest.

## Publisher’s Note

All claims expressed in this article are solely those of the authors and do not necessarily represent those of their affiliated organizations, or those of the publisher, the editors and the reviewers. Any product that may be evaluated in this article, or claim that may be made by its manufacturer, is not guaranteed or endorsed by the publisher.
