# Supplementary material for: Identification and Validation of Three Autophagy-Related Long Noncoding RNAs as Prognostic Signature in Cholangiocarcinoma
Source: Front Oncol. 2021 Dec 2;11:780601. doi: 10.3389/fonc.2021.780601 (PMC8674813; doi:10.3389/fonc.2021.780601)
Supplement: Supplementary file 9 [file Table_1.doc]

| **symbol** | **type** | **numInteractions** |
| --- | --- | --- |
| SLC7A2 | pc | 2 |
| PDK4 | pc | 5 |
| FBXL3 | pc | 2 |
| TSPAN9 | pc | 3 |
| ELOVL5 | pc | 2 |
| CPS1 | pc | 3 |
| CCDC28A | pc | 2 |
| SEC63 | pc | 2 |
| PI4K2B | pc | 2 |
| SCML1 | pc | 3 |
| PER3 | pc | 3 |
| ZNF275 | pc | 6 |
| NTN1 | pc | 2 |
| MYLK | pc | 2 |
| LRRC40 | pc | 2 |
| ISOC1 | pc | 2 |
| ATP1B3 | pc | 1 |
| LRP6 | pc | 6 |
| TESK2 | pc | 5 |
| TEAD2 | pc | 1 |
| MBNL3 | pc | 5 |
| UBE2T | pc | 1 |
| PLEKHG2 | pc | 2 |
| RAPGEF4 | pc | 5 |
| HECTD1 | pc | 4 |
| SEMA6A | pc | 4 |
| MAP3K1 | pc | 7 |
| CREM | pc | 6 |
| GADD45B | pc | 3 |
| RANGAP1 | pc | 1 |
| GNPNAT1 | pc | 2 |
| CNIH1 | pc | 5 |
| SEC23A | pc | 2 |
| RNF24 | pc | 4 |
| MAPRE1 | pc | 6 |
| CDADC1 | pc | 3 |
| PMPCB | pc | 2 |
| TTC26 | pc | 1 |
| ZFAND5 | pc | 5 |
| SLC38A1 | pc | 3 |
| CHPT1 | pc | 2 |
| FANCE | pc | 2 |
| E2F3 | pc | 6 |
| PTK7 | pc | 1 |
| CNOT6 | pc | 7 |
| ARRDC3 | pc | 8 |
| ABCC5 | pc | 6 |
| ERRFI1 | pc | 2 |
| SLC35D1 | pc | 3 |
| GADD45A | pc | 2 |
| TMED5 | pc | 2 |
| KDSR | pc | 2 |
| PPP1R3C | pc | 2 |
| FAM45A | pc | 5 |
| AKAP1 | pc | 5 |
| C16orf70 | pc | 5 |
| SOX9 | pc | 3 |
| TRIP10 | pc | 3 |
| DYRK2 | pc | 6 |
| E2F8 | pc | 2 |
| CBFA2T3 | pc | 2 |
| LDLR | pc | 5 |
| H19 | lnc | 11 |
| PRKAB2 | pc | 2 |
| BHLHE40 | pc | 2 |
| SOX5 | pc | 2 |
| TBX3 | pc | 2 |
| PAN2 | pc | 3 |
| HNRNPA1 | pc | 3 |
| FHOD1 | pc | 1 |
| STX6 | pc | 3 |
| LAMC1 | pc | 6 |
| COG3 | pc | 2 |
| PPP2R1B | pc | 5 |
| MYOF | pc | 1 |
| FAM13A | pc | 5 |
| RASGEF1B | pc | 2 |
| DUSP6 | pc | 2 |
| ANKRD52 | pc | 16 |
| CAPN10 | pc | 1 |
| PLK4 | pc | 1 |
| UBAP2L | pc | 3 |
| IQSEC1 | pc | 2 |
| KLHL8 | pc | 3 |
| PIK3R1 | pc | 5 |
| DAAM2 | pc | 3 |
| PNRC1 | pc | 2 |
| RSPO3 | pc | 2 |
| TACC1 | pc | 6 |
| SYBU | pc | 2 |
| ARHGAP39 | pc | 1 |
| NRBF2 | pc | 5 |
| CTAGE5 | pc | 2 |
| CRIM1 | pc | 6 |
| NUBPL | pc | 2 |
| ACSL1 | pc | 7 |
| PANK1 | pc | 2 |
| RMND5A | pc | 7 |
| NUS1 | pc | 5 |
| ENAH | pc | 6 |
| C16orf87 | pc | 3 |
| ADAMTSL3 | pc | 2 |
| TMEM164 | pc | 1 |
| RER1 | pc | 2 |
| AUTS2 | pc | 2 |
| NIT1 | pc | 1 |
| ATP13A2 | pc | 1 |
| ZNF714 | pc | 1 |
| NFIA | pc | 10 |
| SERINC5 | pc | 3 |
| ADCY1 | pc | 5 |
| ZHX1 | pc | 2 |
| PGM2L1 | pc | 6 |
| PRDX3 | pc | 2 |
| HPRT1 | pc | 3 |
| E2F7 | pc | 6 |
| ARL5B | pc | 2 |
| SPRED1 | pc | 6 |
| GPT2 | pc | 3 |
| MAPRE2 | pc | 3 |
| RFWD3 | pc | 2 |
| SLC25A6 | pc | 2 |
| CCDC126 | pc | 5 |
| TBC1D10B | pc | 3 |
| SMAGP | pc | 1 |
| SNTB1 | pc | 2 |
| CEBPB | pc | 2 |
| ANKRD13D | pc | 1 |
| CKS1B | pc | 1 |
| EXO1 | pc | 1 |
| C4orf32 | pc | 3 |
| KLC2 | pc | 3 |
| ATAD5 | pc | 3 |
| LMNB2 | pc | 2 |
| AQP11 | pc | 2 |
| DHTKD1 | pc | 2 |
| IQGAP3 | pc | 2 |
| IRAK1 | pc | 2 |
| PTP4A3 | pc | 1 |
| INSIG1 | pc | 6 |
| FAM122A | pc | 2 |
| RELN | pc | 2 |
| MYL6B | pc | 1 |
| WNK3 | pc | 5 |
| SLC39A10 | pc | 6 |
| NOL4L | pc | 6 |
| LRP10 | pc | 2 |
| SNHG12 | lnc | 1 |
| NRARP | pc | 9 |
| SH3BGRL2 | pc | 2 |
| PPP1R14C | pc | 4 |
| SMOC1 | pc | 5 |
| PRC1 | pc | 2 |
| RTL8A | pc | 2 |
| LINC00963 | lnc | 1 |
| CHIC1 | pc | 13 |
| ETFRF1 | pc | 2 |
| C1RL-AS1 | lnc | 2 |
| HCG18 | lnc | 8 |
| AL359915.2 | lnc | 1 |
| SNHG15 | lnc | 1 |
| MAGI2-AS3 | lnc | 2 |
| GAS5 | lnc | 1 |
| C12orf75 | pc | 1 |
| ASH1L-AS1 | lnc | 1 |
| SNHG3 | lnc | 1 |
| CEBPA | pc | 2 |
| PVT1 | lnc | 7 |
| SNHG1 | lnc | 4 |
| FBXL19-AS1 | lnc | 9 |
| AC109460.3 | lnc | 3 |
| AC015813.1 | lnc | 1 |

**Table2 (miRNAs)**

| **miRNAs** | **mRNAs** |
| --- | --- |
| hsa-miR-374b-5p | MAGI2-AS3 |
| hsa-miR-374a-5p |  |
| hsa-miR-137 | GAS5 |
| hsa-miR-377-3p | SNHG1 |
| hsa-miR-421 |  |
| hsa-miR-326 |  |
| hsa-miR-330-5p |  |
| hsa-miR-29c-3p | H19 |
| hsa-miR-29b-3p |  |
| hsa-miR-130a-3p |  |
| hsa-miR-454-3p |  |
| hsa-miR-130b-3p |  |
| hsa-miR-29a-3p |  |
| hsa-miR-4295 |  |
| hsa-miR-3666 |  |
| hsa-miR-138-5p |  |
| hsa-miR-107 |  |
| hsa-miR-103a-3p |  |
| hsa-miR-16-5p | HCG18 |
| hsa-miR-15a-5p |  |
| hsa-miR-195-5p |  |
| hsa-miR-497-5p |  |
| hsa-miR-15b-5p |  |
| hsa-miR-424-5p |  |
| hsa-miR-146b-5p |  |
| hsa-miR-146a-5p |  |
| hsa-miR-378f | FBXL19-AS1 |
| hsa-miR-378d |  |
| hsa-miR-378e |  |
| hsa-miR-378c |  |
| hsa-miR-422a |  |
| hsa-miR-378i |  |
| hsa-miR-378b |  |
| hsa-miR-378a-3p |  |
| hsa-miR-378h |  |
| hsa-miR-182-5p | AL359915.2 |
| hsa-miR-125a-3p | LINC00963 |
| hsa-miR-17-5p | PVT1 |
| hsa-miR-20a-5p |  |
| hsa-miR-519d-3p |  |
| hsa-miR-93-5p |  |
| hsa-miR-106b-5p |  |
| hsa-miR-20b-5p |  |
| hsa-miR-106a-5p |  |
| hsa-miR-24-3p | SNHG15 |
| hsa-miR-494-3p |  |
| hsa-miR-16-5p | C1RL-AS1 |
| hsa-miR-424-5p |  |
| hsa-miR-340-5p | SNHG3 |
| hsa-miR-590-3p | AC015813.1 |
| hsa-miR-503-5p | ASH1L-AS1 |
| hsa-miR-761 | AC109460.3 |
| hsa-miR-214-3p |  |
| hsa-miR-3619-5p |  |
| hsa-miR-374b-5p  hsa-miR-374a-5p | TBX3 |
|  | PRDX3 |
|  | TMED5 |
|  | SEC23A |
|  | PMPCB |
|  | CEBPA |
|  | CTAGE5 |
|  | PPP1R3C |
|  | SNTB1 |
|  | GNPNAT1 |
|  | GADD45A |
|  | RER1 |
|  | LRRC40 |
|  | SEC63 |
|  | AUTS2 |
|  | ERRFI1 |
|  | DUSP6 |
|  | ARL5B |
|  | CEBPB |
|  | HECTD1 |
|  | NTN1 |
|  | PNRC1 |
|  | COG3 |
|  | ADAMTSL3 |
|  | FBXL3 |
|  | KDSR |
|  | IQSEC1 |
|  | SYBU |
|  | MYLK |
|  | ELOVL5 |
|  | BHLHE40 |
| hsa-miR-137 | EXO1 |
|  | PTP4A3 |
|  | TTC26 |
| hsa-miR-377-3p | E2F8 |
| hsa-miR-421 |  |
| hsa-miR-326 | HNRNPA1 |
| hsa-miR-330-5p |  |
| hsa-miR-421 |  |
| hsa-miR-326 | LMNB2 |
| hsa-miR-330-5p |  |
| hsa-miR-326 | RTL8A |
| hsa-miR-330-5p |  |
| hsa-miR-326 | RFWD3 |
| hsa-miR-330-5p |  |
| hsa-miR-326 | FANCE |
| hsa-miR-330-5p |  |
| hsa-miR-29c-3p | INSIG1 |
| hsa-miR-29b-3p |  |
| hsa-miR-130a-3p |  |
| hsa-miR-454-3p |  |
| hsa-miR-130b-3p |  |
| hsa-miR-29a-3p |  |
| hsa-miR-4295 | ACSL1 |
| hsa-miR-130a-3p |  |
| hsa-miR-454-3p |  |
| hsa-miR-130b-3p |  |
| hsa-miR-3666 |  |
| hsa-miR-29c-3p | CPS1 |
| hsa-miR-29b-3p |  |
| hsa-miR-29a-3p |  |
| hsa-miR-4295 | FAM13A |
| hsa-miR-130a-3p |  |
| hsa-miR-454-3p |  |
| hsa-miR-130b-3p |  |
| hsa-miR-3666 |  |
| hsa-miR-4295 | C16orf70 |
| hsa-miR-130a-3p |  |
| hsa-miR-454-3p |  |
| hsa-miR-130b-3p |  |
| hsa-miR-3666 |  |
| hsa-miR-4295 | RAPGEF4 |
| hsa-miR-130a-3p |  |
| hsa-miR-454-3p |  |
| hsa-miR-130b-3p |  |
| hsa-miR-3666 |  |
| hsa-miR-4295 | WNK3 |
| hsa-miR-130a-3p |  |
| hsa-miR-454-3p |  |
| hsa-miR-130b-3p |  |
| hsa-miR-3666 |  |
| hsa-miR-130a-3p | SLC35D1 |
| hsa-miR-454-3p |  |
| hsa-miR-130b-3p |  |
| hsa-miR-4295 | SMOC1 |
| hsa-miR-130a-3p |  |
| hsa-miR-454-3p |  |
| hsa-miR-130b-3p |  |
| hsa-miR-3666 |  |
| hsa-miR-4295 | NRBF2 |
| hsa-miR-130a-3p |  |
| hsa-miR-454-3p |  |
| hsa-miR-130b-3p |  |
| hsa-miR-3666 |  |
| hsa-miR-4295 | PPP2R1B |
| hsa-miR-130a-3p |  |
| hsa-miR-454-3p |  |
| hsa-miR-130b-3p |  |
| hsa-miR-3666 |  |
| hsa-miR-130a-3p | GPT2 |
| hsa-miR-454-3p |  |
| hsa-miR-130b-3p |  |
| hsa-miR-29c-3p | RMND5A |
| hsa-miR-29b-3p |  |
| hsa-miR-130a-3p |  |
| hsa-miR-138-5p |  |
| hsa-miR-454-3p |  |
| hsa-miR-130b-3p |  |
| hsa-miR-29a-3p |  |
| hsa-miR-4295 | ZFAND5 |
| hsa-miR-130a-3p |  |
| hsa-miR-454-3p |  |
| hsa-miR-130b-3p |  |
| hsa-miR-3666 |  |
| hsa-miR-130a-3p | CDADC1 |
| hsa-miR-454-3p |  |
| hsa-miR-130b-3p |  |
| hsa-miR-4295 | ADCY1 |
| hsa-miR-130a-3p |  |
| hsa-miR-454-3p |  |
| hsa-miR-130b-3p |  |
| hsa-miR-3666 |  |
| hsa-miR-4295 | CNIH1 |
| hsa-miR-130a-3p |  |
| hsa-miR-454-3p |  |
| hsa-miR-130b-3p |  |
| hsa-miR-3666 |  |
| hsa-miR-29c-3p | PER3 |
| hsa-miR-29b-3p |  |
| hsa-miR-29a-3p |  |
| hsa-miR-29c-3p | PIK3R1 |
| hsa-miR-29b-3p |  |
| hsa-miR-107 |  |
| hsa-miR-103a-3p |  |
| hsa-miR-29a-3p |  |
| hsa-miR-29c-3p  hsa-miR-29b-3p  hsa-miR-29a-3p | C16orf87 |
|  | TSPAN9 |
| hsa-miR-4295 | ZNF275 |
| hsa-miR-130a-3p |  |
| hsa-miR-138-5p |  |
| hsa-miR-454-3p |  |
| hsa-miR-130b-3p |  |
| hsa-miR-3666 |  |
| hsa-miR-130a-3p | GADD45B |
| hsa-miR-454-3p |  |
| hsa-miR-130b-3p |  |
| hsa-miR-29c-3p  hsa-miR-29b-3p  hsa-miR-29a-3p | SCML1 |
|  | PAN2 |
| hsa-miR-29c-3p | PDK4 |
| hsa-miR-29b-3p |  |
| hsa-miR-107 |  |
| hsa-miR-103a-3p |  |
| hsa-miR-29a-3p |  |
| hsa-miR-29c-3p | NFIA |
| hsa-miR-29b-3p |  |
| hsa-miR-107 |  |
| hsa-miR-4295 |  |
| hsa-miR-130a-3p |  |
| hsa-miR-454-3p |  |
| hsa-miR-103a-3p |  |
| hsa-miR-130b-3p |  |
| hsa-miR-3666 |  |
| hsa-miR-29a-3p |  |
| hsa-miR-4295 | TESK2 |
| hsa-miR-130a-3p |  |
| hsa-miR-454-3p |  |
| hsa-miR-130b-3p |  |
| hsa-miR-3666 |  |
| hsa-miR-29c-3p | CREM |
| hsa-miR-29b-3p |  |
| hsa-miR-130a-3p |  |
| hsa-miR-454-3p |  |
| hsa-miR-130b-3p |  |
| hsa-miR-29a-3p |  |
| hsa-miR-4295 | MBNL3 |
| hsa-miR-130a-3p |  |
| hsa-miR-454-3p |  |
| hsa-miR-130b-3p |  |
| hsa-miR-3666 |  |
| hsa-miR-4295 | FAM45A |
| hsa-miR-130a-3p |  |
| hsa-miR-454-3p |  |
| hsa-miR-130b-3p |  |
| hsa-miR-3666 |  |
| hsa-miR-29c-3p | SERINC5 |
| hsa-miR-29b-3p |  |
| hsa-miR-29a-3p |  |
| hsa-miR-29b-3p | NUBPL |
| hsa-miR-29a-3p |  |
| hsa-miR-4295 | NUS1 |
| hsa-miR-130a-3p |  |
| hsa-miR-454-3p |  |
| hsa-miR-130b-3p |  |
| hsa-miR-3666 |  |
| hsa-miR-4295 | AKAP1 |
| hsa-miR-130a-3p |  |
| hsa-miR-454-3p |  |
| hsa-miR-130b-3p |  |
| hsa-miR-3666 |  |
| hsa-miR-29c-3p | MAPRE2 |
| hsa-miR-29b-3p |  |
| hsa-miR-29a-3p |  |
| hsa-miR-4295 | CCDC126 |
| hsa-miR-130a-3p |  |
| hsa-miR-454-3p |  |
| hsa-miR-130b-3p |  |
| hsa-miR-3666 |  |
| hsa-miR-29c-3p | LRP6 |
| hsa-miR-29b-3p |  |
| hsa-miR-130a-3p |  |
| hsa-miR-454-3p |  |
| hsa-miR-130b-3p |  |
| hsa-miR-29a-3p |  |
| hsa-miR-29c-3p | KLHL8 |
| hsa-miR-29b-3p |  |
| hsa-miR-29a-3p |  |
| hsa-miR-130a-3p | HPRT1 |
| hsa-miR-454-3p |  |
| hsa-miR-130b-3p |  |
| hsa-miR-29c-3p | C4orf32 |
| hsa-miR-29b-3p |  |
| hsa-miR-29a-3p |  |
| hsa-miR-4295 | LDLR |
| hsa-miR-130a-3p |  |
| hsa-miR-454-3p |  |
| hsa-miR-130b-3p |  |
| hsa-miR-3666 |  |
| hsa-miR-29c-3p | ARRDC3 |
| hsa-miR-29b-3p |  |
| hsa-miR-4295 |  |
| hsa-miR-130a-3p |  |
| hsa-miR-454-3p |  |
| hsa-miR-130b-3p |  |
| hsa-miR-3666 |  |
| hsa-miR-29a-3p |  |
| hsa-miR-29c-3p | DAAM2 |
| hsa-miR-29b-3p |  |
| hsa-miR-29a-3p |  |
| hsa-miR-16-5p | MAPRE1 |
| hsa-miR-15a-5p |  |
| hsa-miR-195-5p |  |
| hsa-miR-497-5p |  |
| hsa-miR-15b-5p |  |
| hsa-miR-424-5p |  |
| hsa-miR-146b-5p | IRAK1 |
| hsa-miR-146a-5p |  |
| hsa-miR-16-5p | LAMC1 |
| hsa-miR-15a-5p |  |
| hsa-miR-195-5p |  |
| hsa-miR-497-5p |  |
| hsa-miR-15b-5p |  |
| hsa-miR-424-5p |  |
| hsa-miR-16-5p | E2F7 |
| hsa-miR-15a-5p |  |
| hsa-miR-195-5p |  |
| hsa-miR-497-5p |  |
| hsa-miR-15b-5p |  |
| hsa-miR-424-5p |  |
| hsa-miR-16-5p | SLC39A10 |
| hsa-miR-15a-5p |  |
| hsa-miR-195-5p |  |
| hsa-miR-497-5p |  |
| hsa-miR-15b-5p |  |
| hsa-miR-424-5p |  |
| hsa-miR-15a-5p | LRP10 |
| hsa-miR-15b-5p |  |
| hsa-miR-146b-5p | PRC1 |
| hsa-miR-146a-5p |  |
| hsa-miR-16-5p | DYRK2 |
| hsa-miR-15a-5p |  |
| hsa-miR-195-5p |  |
| hsa-miR-497-5p |  |
| hsa-miR-15b-5p |  |
| hsa-miR-424-5p |  |
| hsa-miR-16-5p | ATAD5 |
| hsa-miR-195-5p |  |
| hsa-miR-15b-5p |  |
| hsa-miR-15a-5p | SOX9 |
| hsa-miR-15b-5p |  |
| hsa-miR-424-5p |  |
| hsa-miR-146b-5p | IQGAP3 |
| hsa-miR-146a-5p |  |
| hsa-miR-16-5p | ENAH |
| hsa-miR-15a-5p |  |
| hsa-miR-195-5p |  |
| hsa-miR-497-5p |  |
| hsa-miR-15b-5p |  |
| hsa-miR-424-5p |  |
| hsa-miR-16-5p | ABCC5 |
| hsa-miR-15a-5p |  |
| hsa-miR-195-5p |  |
| hsa-miR-497-5p |  |
| hsa-miR-15b-5p |  |
| hsa-miR-424-5p |  |
| hsa-miR-15a-5p | PPP1R14C |
| hsa-miR-497-5p |  |
| hsa-miR-15b-5p |  |
| hsa-miR-424-5p |  |
| hsa-miR-16-5p | TACC1 |
| hsa-miR-15a-5p |  |
| hsa-miR-195-5p |  |
| hsa-miR-497-5p |  |
| hsa-miR-15b-5p |  |
| hsa-miR-424-5p |  |
| hsa-miR-16-5p | NOL4L |
| hsa-miR-15a-5p |  |
| hsa-miR-195-5p |  |
| hsa-miR-497-5p |  |
| hsa-miR-15b-5p |  |
| hsa-miR-424-5p |  |
| hsa-miR-16-5p | PGM2L1 |
| hsa-miR-15a-5p |  |
| hsa-miR-195-5p |  |
| hsa-miR-497-5p |  |
| hsa-miR-15b-5p |  |
| hsa-miR-424-5p |  |
| hsa-miR-16-5p | SPRED1 |
| hsa-miR-15a-5p |  |
| hsa-miR-195-5p |  |
| hsa-miR-497-5p |  |
| hsa-miR-15b-5p |  |
| hsa-miR-424-5p |  |
| hsa-miR-16-5p | NRARP |
| hsa-miR-15a-5p |  |
| hsa-miR-195-5p |  |
| hsa-miR-497-5p |  |
| hsa-miR-15b-5p |  |
| hsa-miR-424-5p |  |
| hsa-miR-16-5p | E2F3 |
| hsa-miR-15a-5p |  |
| hsa-miR-195-5p |  |
| hsa-miR-497-5p |  |
| hsa-miR-15b-5p |  |
| hsa-miR-424-5p |  |
| hsa-miR-16-5p | CRIM1 |
| hsa-miR-15a-5p |  |
| hsa-miR-195-5p |  |
| hsa-miR-497-5p |  |
| hsa-miR-15b-5p |  |
| hsa-miR-424-5p |  |
| hsa-miR-16-5p | CHIC1 |
| hsa-miR-15a-5p |  |
| hsa-miR-195-5p |  |
| hsa-miR-497-5p |  |
| hsa-miR-15b-5p |  |
| hsa-miR-424-5p |  |
| hsa-miR-16-5p | SEMA6A |
| hsa-miR-15a-5p |  |
| hsa-miR-195-5p |  |
| hsa-miR-15b-5p |  |
| hsa-miR-378f | SLC38A1 |
| hsa-miR-378d |  |
| hsa-miR-378e |  |
| hsa-miR-378f | ANKRD52 |
| hsa-miR-378c |  |
| hsa-miR-422a |  |
| hsa-miR-378i |  |
| hsa-miR-378b |  |
| hsa-miR-378d |  |
| hsa-miR-378a-3p |  |
| hsa-miR-378h |  |
| hsa-miR-378e |  |
| hsa-miR-422a | SLC25A6 |
| hsa-miR-378a-3p |  |
| hsa-miR-422a | PLEKHG2 |
| hsa-miR-378a-3p |  |
| hsa-miR-182-5p | UBE2T |
| hsa-miR-182-5p | ATP1B3 |
| hsa-miR-125a-3p | NIT1 |
| hsa-miR-17-5p | ANKRD52 |
| hsa-miR-20a-5p |  |
| hsa-miR-519d-3p |  |
| hsa-miR-93-5p |  |
| hsa-miR-106b-5p |  |
| hsa-miR-20b-5p |  |
| hsa-miR-106a-5p |  |
| hsa-miR-17-5p | CNOT6 |
| hsa-miR-20a-5p |  |
| hsa-miR-519d-3p |  |
| hsa-miR-93-5p |  |
| hsa-miR-106b-5p |  |
| hsa-miR-20b-5p |  |
| hsa-miR-106a-5p |  |
| hsa-miR-17-5p | RNF24 |
| hsa-miR-20a-5p |  |
| hsa-miR-93-5p |  |
| hsa-miR-20b-5p |  |
| hsa-miR-17-5p | MAP3K1 |
| hsa-miR-20a-5p |  |
| hsa-miR-519d-3p |  |
| hsa-miR-93-5p |  |
| hsa-miR-106b-5p |  |
| hsa-miR-20b-5p |  |
| hsa-miR-106a-5p |  |
| hsa-miR-17-5p | CHIC1 |
| hsa-miR-20a-5p |  |
| hsa-miR-519d-3p |  |
| hsa-miR-93-5p |  |
| hsa-miR-106b-5p |  |
| hsa-miR-20b-5p |  |
| hsa-miR-106a-5p |  |
| hsa-miR-24-3p | ATP13A2 |
| hsa-miR-24-3p | ARHGAP39 |
| hsa-miR-24-3p | SMAGP |
| hsa-miR-494-3p | TMEM164 |
| hsa-miR-494-3p | MYOF |
| hsa-miR-494-3p | CKS1B |
| hsa-miR-494-3p | MYL6B |
| hsa-miR-16-5p | ETFRF1 |
| hsa-miR-424-5p |  |
| hsa-miR-16-5p | ISOC1 |
| hsa-miR-424-5p |  |
| hsa-miR-16-5p | SOX5 |
| hsa-miR-424-5p |  |
| hsa-miR-16-5p | DHTKD1 |
| hsa-miR-424-5p |  |
| hsa-miR-16-5p | AQP11 |
| hsa-miR-424-5p |  |
| hsa-miR-16-5p | PANK1 |
| hsa-miR-424-5p |  |
| hsa-miR-16-5p | PI4K2B |
| hsa-miR-424-5p |  |
| hsa-miR-16-5p | ACSL1 |
| hsa-miR-424-5p |  |
| hsa-miR-16-5p | RASGEF1B |
| hsa-miR-424-5p |  |
| hsa-miR-16-5p | SLC7A2 |
| hsa-miR-424-5p |  |
| hsa-miR-16-5p | CHPT1 |
| hsa-miR-424-5p |  |
| hsa-miR-16-5p | CCDC28A |
| hsa-miR-424-5p |  |
| hsa-miR-16-5p | FAM122A |
| hsa-miR-424-5p |  |
| hsa-miR-16-5p | RELN |
| hsa-miR-424-5p |  |
| hsa-miR-16-5p | ZHX1 |
| hsa-miR-424-5p |  |
| hsa-miR-16-5p | RSPO3 |
| hsa-miR-424-5p |  |
| hsa-miR-16-5p | CBFA2T3 |
| hsa-miR-424-5p |  |
| hsa-miR-16-5p | HECTD1 |
| hsa-miR-424-5p |  |
| hsa-miR-16-5p | SH3BGRL2 |
| hsa-miR-424-5p |  |
| hsa-miR-16-5p | PRKAB2 |
| hsa-miR-424-5p |  |
| hsa-miR-340-5p | PLK4 |
| hsa-miR-340-5p | TEAD2 |
| hsa-miR-340-5p | ANKRD13D |
| hsa-miR-340-5p | CAPN10 |
| hsa-miR-340-5p | C12orf75 |
| hsa-miR-590-3p | FHOD1 |
| hsa-miR-590-3p | ZNF714 |
| hsa-miR-503-5p | RANGAP1 |
| hsa-miR-503-5p | PTK7 |
| hsa-miR-761 | KLC2 |
| hsa-miR-214-3p |  |
| hsa-miR-3619-5p |  |
| hsa-miR-761 | TBC1D10B |
| hsa-miR-214-3p |  |
| hsa-miR-3619-5p |  |
| hsa-miR-761 | TRIP10 |
| hsa-miR-214-3p |  |
| hsa-miR-3619-5p |  |
| hsa-miR-761 | UBAP2L |
| hsa-miR-214-3p |  |
| hsa-miR-3619-5p |  |
| hsa-miR-761 | STX6 |
| hsa-miR-214-3p |  |
| hsa-miR-3619-5p |  |
| hsa-miR-761 | NRARP |
| hsa-miR-214-3p |  |
| hsa-miR-3619-5p |  |
